# Efficient Text Encryption and Hiding with Double-Random Phase-Encoding

**DOI:** 10.3390/s121013441

**Published:** 2012-10-01

**Authors:** Jun Sang, Shenggui Ling, Mohammad S. Alam

**Affiliations:** 1 School of Software Engineering, Chongqing University, Chongqing 401331, China; E-Mail: 20092402023@cqu.edu.cn; 2 Department of Electrical and Computer Engineering, University of South Alabama, Mobile, AL 36688, USA; E-Mail: malam@southalabama.edu

**Keywords:** double-random phase-encoding, text encryption, information hiding, hiding capacity, recovery accuracy

## Abstract

In this paper, a double-random phase-encoding technique-based text encryption and hiding method is proposed. First, the secret text is transformed into a 2-dimensional array and the higher bits of the elements in the transformed array are used to store the bit stream of the secret text, while the lower bits are filled with specific values. Then, the transformed array is encoded with double-random phase-encoding technique. Finally, the encoded array is superimposed on an expanded host image to obtain the image embedded with hidden data. The performance of the proposed technique, including the hiding capacity, the recovery accuracy of the secret text, and the quality of the image embedded with hidden data, is tested via analytical modeling and test data stream. Experimental results show that the secret text can be recovered either accurately or almost accurately, while maintaining the quality of the host image embedded with hidden data by properly selecting the method of transforming the secret text into an array and the superimposition coefficient. By using optical information processing techniques, the proposed method has been found to significantly improve the security of text information transmission, while ensuring hiding capacity at a prescribed level.

## 1. Introduction

In information security, cryptography, which encrypts the secret message before transmission to avoid information disclosure, is commonly used [1]. Usually, the encryption methods are based on digital methods [1]. By utilizing the high processing speed, high parallelism, and high-dimensional encryption characteristics of the optical information processing system, optical encryption methods outperform digital encryption methods for image encryption. A typical optical image encryption method is the double-random phase-encoding (DRPE) technique, which encodes the original secret image to a complex-valued encoded image by applying independent random phase encoding on the input plane and the Fourier plane, respectively [2]. Thereafter, the DRPE-based optical image encryption method has been improved [3–7] and applied to other transformation domains, including optical encryption in the fractional Fourier domain [8–12], optical encryption in the Fresnel domain [13,14] and encrypting information with digital holography [15,16].

For cryptography, the encrypted secret message, *i.e.*, the cyphertext, is usually unreadable. Therefore, an encrypted message may be easily detected during transmission on the public channel, which will disclose the existence of the secret transmission. On the other hand, information hiding techniques hide the secret message in public information to conceal the existence of the secret message and to achieve secure message storage and transmission [17]. Information hiding includes two main types [17]: (1) watermarking, which is usually used to protect the copyright of digital products or used to ensure the authenticity and integrity of the digital content; (2) steganorgraphy, which is usually used for secure transmissions.

The traditional information hiding methods usually use digital methods to hide the secret message in the spatial domain [18,19] or in the frequency domain [20–25]. Recently, optical information processing techniques were used for information hiding, including hiding images in halftone pictures [26], double-random phase-encoding [27,28], digital holography [29–33], Fresnel domain [34,35] and hybrid methods [36–38]. To enhance security, an information hiding technique is usually combined with encryption to encrypt the secret message before hiding it in the public information [39]. As a typical optical image encryption technique, the DRPE technique has been widely used to hide the secret image [27,28,40,41].

The existing DRPE-based information hiding methods are usually used for image hiding [28,40,41]. They are also employed to encrypt and hide the secret text in this paper. First, the secret text is transformed into a 2-dimensional array in the form of an image. The bit stream of the secret text is stored in the higher bits of the elements of the transformed array, while the lower bits of the elements are filled with specific values. Then, the transformed array is encoded with the DRPE technique and is hidden into an expanded host image. To recover the secret text, the encoded array is extracted from the image embedded with hidden data and decrypted with the DRPE technique. Thereafter, the bit stream of the secret text is obtained from the higher bits of the elements in the decrypted array. Thus, by using this bit stream, the secret text can be recovered. In the proposed method, the DRPE-based image encryption and decryption may be realized with optical method and the other steps may be realized with digital methods. The proposed method applies the optical information encryption and hiding method to text encryption and hiding, which increases the security of the secret text while utilizing the advantages of optical information processing techniques.

The rest of this paper is organized as follows: in Section 2, the proposed method is introduced. Section 3 incorporates the theoretical performance analysis of the proposed method, including the hiding capacity, the recovery accuracy of the secret text, and the quality of the image embedded with hidden data. Section 4 includes the results and discussions obtained from the numerical simulation experiments. Section 5 presents the concluding remarks.

## 2. The Proposed Scheme

In this Section, the proposed text encryption and hiding method based on DRPE technique is introduced. The main symbols used in this paper are listed in Table 1.

### 2.1. Encoding and Hiding Procedure

**Figure 1.**
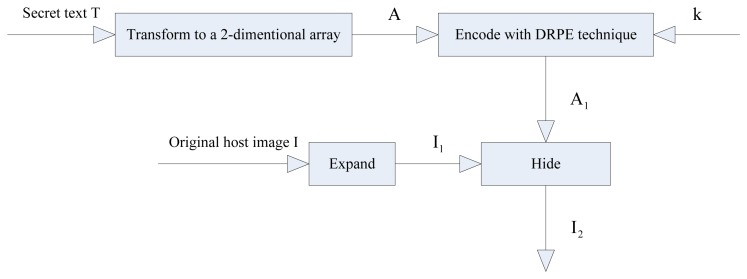
Encoding and hiding procedure.

The encoding and hiding procedure used in this paper is shown in the block diagram of Figure 1.

In the encoding and hiding procedure, at first, the secret text is transformed into a 2-dimensional array. Then, the transformed array is encoded with the DRPE technique and the encoded array is used to construct the hidden data array. Finally, the hidden data array is hidden into the expanded host image with superimposition. The detailed steps involved in this process are described as follows:

Step 1: Transform the secret text into a 2-dimensional array.
  In this step, the secret text is transformed into a 2-dimentional array in the form of an image to encode with the DRPE technique. To transform the secret text *T* into a 2-dimentional array *A*, *T* is denoted as a bit stream. The bit stream is stored in the higher bits of the elements in *A*, while the lower bits of the elements in *A* are filled with 0 s or 1 s. Each element in *A* is an integer with a value ranging from 0 and 255 corresponding to 8 bits, which is the same as the pixel value in an 8-bit grayscale image. Thus, the transformed array *A* can be viewed as a grayscale image. The main reason for using the higher bits instead of the entire set of available bits to store the bit stream of the secret text *T* is to ensure the recovery accuracy of the secret text, since the DRPE-based information hiding method inherently generates computational errors [28,41]. Assume the secret text *T* with *L* ASCII codes is transformed into a 2-dimensional array *A* with a size of *M* × *N* pixels and the number of the higher bits used to store the bit stream of *T* is m. Therefore, at least $\frac{8\times L}{m}$ elements in *A* will be needed to store the bit stream of T, *i.e.*, $(M\times N)\geq \frac{8\times L}{m}$.
Step 2: Encode the transformed array with the DRPE technique.
  In this step, the transformed array is encoded by using the DRPE technique [2]. The transformed array *A* with a size of *M* × *N* pixels can be encoded with the DRPE technique to obtain a 2-dimensional complex array *A*_1_ with a size of *M* × *N* pixels as:
  (1) $$A_{1}(x,y)=FT^{-1}\{FT\{A(x,y)\exp[j2\pi n(x,y)]\}\exp[j2\pi b(\xi ,\eta )]\}$$
  where *n*(*x,y*) and *b*(*ξ*,*η*) denote two independent random functions, which are uniformly distributed with a range of 0 to 1 and can be taken as the secret key *k* for encoding. They can be created with the existing random sequence generation methods [1] by using a software, such as MATLAB, with different parameters. *FT* and *FT^-1^* represent the Fourier transform and inverse Fourier transform, respectively. Assume that the encoded complex-valued array *A*_1_ is defined as:
  (2) $$A_{1}=A_{1R}+jA_{1I}$$
  where *A*_1_*_R_* and *A*_1_*_I_* are the real part and the imaginary part of *A*_1_, respectively. Both of these arrays are with size of *M* × *N* pixels.
Step 3: Construct the hidden data array.
  To hide the encoded complex-valued array into the host image, in this step, the hidden data array is constructed from *A*_1_. There are various ways to construct the hidden data array, which may result in different hiding capacities [28,41]. In reference [28], one element in the real part of *A*_1_, *i.e.*, *A*_1_*_R_*, and the corresponding element in the imaginary part of *A*_1_, *i.e.*, *A*_1_*_I_*, were used to construct a 2 × 2 subarray, expressed as:
  (3) $$\left[\begin{array}{rr} a1 & -a2\\ a2 & -a1 \end{array}\right]$$
  where *a*1 is the element in *A*_1_*_R_*, while *a*2 is the corresponding element in *A*_1_*_I_*. The hidden data array is composed of these 2 × 2 subarrays. In reference [41], two elements in *A*_1_*_R_* and one element in *A*_1_*_I_* are used sequentially, or one element in *A*_1_*_R_* and two elements in *A*_1_*_I_* are used sequentially, to construct the 2 × 2 subarrays, expressed as:
  (4) $$\left[\begin{array}{ll} a1 & a2\\ a3 & 0 \end{array}\right]$$
  where *a*1, *a*2 and *a*3 are the elements taken from *A*_1_*_R_* or *A*_1_*_I_*. The hidden data array is composed of these 2 × 2 arrays.
Step 4: Expand the host image.
  In this step, the original host image is expanded to hide the hidden data array, which is constructed using the above mentioned procedure. The original host image *I* with a size of *M_1_* × *N_1_* pixels is expanded to form another image *I*_1_ with a size of 2*M_1_* × *2N_1_* pixels by expanding each pixel into a 2 × 2 subarray, such that:
  (5) $$\{\begin{array}{l} I_{1}(2x,2y)=I(x,y)\\ I_{1}(2x,2y+1)=I(x,y)\\ I_{1}(2x+1,2y)=I(x,y)\\ I_{1}(2x+1,2y+1)=I(x,y) \end{array}$$
  where *x* = 0,1,2,…, *M*_1_–1, and y = 0,1,2,…, *N*_1_–1, respectively. According to the procedure of reference [28], to hide the encoded complex-valued *M* × *N* array *A*_1_, a total of MN pixels are needed (*i.e.*, *M*_1_ = *M* and *N*_1_ = *N*). However, according to the procedure of reference [41], since three values can be hidden in a 2 × 2 subarray, when $M_{1}=\left\lceil \sqrt{\frac{2}{3}}\cdot M\right\rceil$ and $N_{1}=\left\lceil \sqrt{\frac{2}{3}}\cdot N\right\rceil$, a total of *M*_1_ *N*_1_ pixels are enough to hide *A*_1_, where ⌈ ● ⌉ denotes the ceiling operation.
Step 5: Hide the constructed hidden data array into the expanded host image.
  In this step, the constructed hidden data array is hidden into the expanded host image *I*_1_ by superimposing each 2 × 2 subarray in the hidden data array into the corresponding 2 × 2 subarray in *I*_1_ by processing one pixel at a time as shown below:
  (6) $$\left[\begin{array}{ll} c1+\alpha \cdot a1 & c1-\alpha \cdot a2\\ c3+\alpha \cdot a2 & c4-\alpha \cdot a1 \end{array}\right]$$
  or:
  (7) $$\left[\begin{array}{ll} c1+\alpha \cdot a1 & c2-\alpha \cdot a2\\ c3+\alpha \cdot a3 & c4 \end{array}\right]$$
  where α is the superimposition coefficient, which corresponds to the embedding strength. Equations (6) and (7) are used in conjunction with Equations (3) and (4), respectively, depending on the method used to construct the hidden data array in Step 3.
  As mentioned in Step 4, *c*1, *c*2, *c*3 and *c*4 are obtained by expanding each pixel of the original host image *I* into a 2 × 2 subarray, *i.e.*, they are identical. By hiding the constructed hidden data array into the expanded host image *I*_1_, the image embedded with hidden data *I*_2_ is obtained.

### 2.2. Extraction and Recovery Procedure

**Figure 2.**
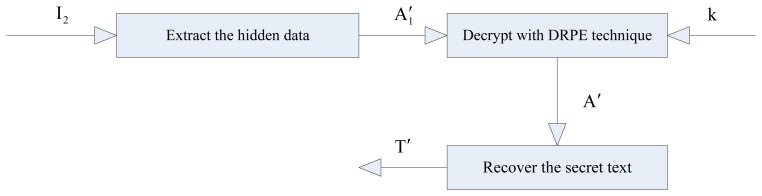
Extraction and recovery procedure.

The extraction and recovery procedure for the proposed technique is shown in Figure 2.

To recover the secret text from image *I*_2_, which is embedded with the hidden data, first, the hidden data array is extracted from *I*_2_ to reconstruct the encoded array *A*_1_′. Then, *A*_1_′ is decrypted by using the DRPE technique to obtain the decrypted array *A*′. Finally, the secret text *T*′ is recovered from *A*′. This procedure involves the following steps:

Step 1: Extract the hidden data array from the image embedded with the hidden data.
  Depending on whether the hidden data array is constructed with the procedure of reference [28] or that of reference [41], it is easy to extract *a*1 and *a*2 from *I*_2_ using Equations (3) and (6) or extract *a*1, *a*2 and *a*3 from *I*_2_ using Equations (4) and (7), respectively. Due to the inherent computational error, the extracted data may vary slightly from the hidden data.
Step 2: Reconstruct the encoded array.
  The data extracted in Step 1 corresponds to the real part or the imaginary part of the complex array encoded with the DRPE technique. With the extracted data, a 2-dimensional complex array *A*_1_′ with a size of *M* × *N* pixels can be reconstructed, which corresponds to the encoded array *A*_1_. Due to computational errors, *A*_1_′ and *A*_1_ may have slight variations.
Step 3: Decrypt the reconstructed array *A*_1_′.
  By decrypting the reconstructed 2-dimensional complex array *A*_1_′ with the DRPE technique, an array *A*′ can be obtained, which corresponds to the transformed array *A*, expressed as:
  (8) $$A^{\prime }(x,y)=FT^{-1}\{FT[A_{1}^{\prime }(x,y)]\exp[-j2\pi b(\xi ,\eta )]\}\exp[-2\pi n(x,y)]$$
  Due to computational errors, *A*′ and *A* may have slight variations.
Step 4: Recover the secret text.
  In this step, the secret text *T*′ is recovered from the transformed array *A*′ obtained in Step 3 by applying the inverse operation of Step 1 in Section 2.1. Due to computational errors, the recovered secret text *T*′ may have slight variation from the original secret text *T*. To recover the secret text accurately, the value of *m*, the method used to fill the lower bits and the value of the superimposition coefficient α should be selected carefully, which are discussed in detail in the following sections.

## 3. Performance Analysis

In this Section, the performance of the proposed method is investigated by using three criteria, *i.e.*, hiding capacity, recovery accuracy of the secret text, and the quality of the image embedded with hidden data.

### 3.1. Hiding Capacity

The hiding capacity is defined as the number of the bytes of the secret text *T* being hidden in a pixel of the image embedded with the hidden data *I*_2_. The hiding capacity is directly related to the number of the higher bits of the elements in the transformed array *A* to be used for storing the bit stream of the secret text *T*, and the method of constructing the hidden data array. In a 2-dimensiontal transformed array *A* with a size of *M* × *N*, assuming that *m* higher bits of the elements are used to store the bit stream of the secret text *T*, $\left\lfloor \frac{M\times N\times m}{8}\right\rfloor$ bytes of secret text can be stored in *A*, where ⌊ ● ⌋ represents the floor operation. If the hidden data array is constructed according to Equation (3), an array *A* with a size of *M* × *N* pixels can be hidden into an expanded host image with a size of 2*M* × 2*N* pixels. Then, the hiding capacity will be equal to $\frac{\left\lfloor \frac{M\times N\times m}{8}\right\rfloor }{2M\times 2N}$. On the other hand, if the hidden data array is constructed according to Equation (4), an array *A* with a size of *M* × *N* can be hidden into an expanded host image with a size of $2\times \left\lceil \sqrt{\frac{2}{3}}\cdot M\right\rceil \times 2\times \left\lceil \sqrt{\frac{2}{3}}\cdot N\right\rceil$ pixels. Then, the hiding capacity becomes equal to $\frac{\left\lfloor \frac{M\times N\times m}{8}\right\rfloor }{2\times \left\lceil \sqrt{\frac{2}{3}}\cdot M\right\rceil \times 2\times \left\lceil \sqrt{\frac{2}{3}}\cdot N\right\rceil }$, where the numerator $\left\lfloor \frac{M\times N\times m}{8}\right\rfloor$ represents the total bytes of the secret text being hidden and the denominator $2\times \left\lceil \sqrt{\frac{2}{3}}\cdot M\right\rceil \times 2\times \left\lceil \sqrt{\frac{2}{3}}\cdot N\right\rceil$ represents the total pixels being needed to hide the secret text.

### 3.2. Recovery Accuracy of the Secret Text

In this paper, the DRPE technique is used to encrypt and hide the desired secret text. The main objective is to accurately recover the hidden secret text. Assume that each character in the secret text is represented by one byte. To assess the secret text recovery result, the recovered secret text *T*′ is compared with the original secret text *T* via byte-by-byte comparison instead of bit-by-bit comparison. Assume that the length of the secret text *T* is *L* bytes, and the number of the accurately recovered bytes is *L*′. The recovery accuracy (*γ*) of the secret text is defined as:

(9) $$\gamma =\frac{L^{\prime }}{L}$$

The recovery accuracy of the secret text is related to the following parameters:

- The number of higher bits of the elements in the transformed array *A* to be used for storing the bit stream of the secret text *T*. Due to the computational error inherent in the DRPE-based encoding and hiding technique, the recovered secret text may be slightly different from the original text. The greater the number (*m*) of higher bits of the elements in the transformed array *A* used to store the secret text *T*, the lower the recovery accuracy. To trade off the hiding capacity and the recovery accuracy of the secret text, the value of *m* must be considered carefully.
- The method used to fill the lower bits of the elements in the transformed array *A*. Note that the bit stream of the secret text *T* is stored in the higher bits of the elements in *A*. To recover the hidden secret text accurately, we may only consider how to accurately recover the higher bits of the elements in *A* instead of the lower bits. On the other hand, according to the characteristics of the DRPE-based information hiding method [28,41], the values of the elements in the decrypted array *A*′ are usually not very different from those of the original transformed array *A*. By properly selecting the method used to fill the lower bits of the elements in *A*, *i.e.*, setting the values of the lower bits properly, the difference between *A*′ and *A* will only influence the lower bits of the elements, while keeping the higher bits of the elements invariant. Therefore, to ensure the recovery accuracy of the secret text, the method used to fill the lower bits of the elements in *A* also needs to be considered carefully. It will be discussed with simulation experiments in the next Section.
- The value of the superimposition coefficient α. As discussed in references [28] and [41], the value of the superimposition coefficient α also influences the decrypted array *A*′, which will influence the recovery accuracy of the secret text. The greater the value of α is, the higher the recovery accuracy.

### 3.3. Quality of the Image Embedded with Hidden Data

The quality of the image embedded with hidden data *I*_2_ is directly related to the value of the superimposition coefficient α. The less the value of α is, the higher the quality of *I*_2_. To trade off the recovery accuracy of the secret text and the quality of *I*_2_, the value of α must be considered carefully.

## 4. Experimental Results and Discussion

**Figure 3.**
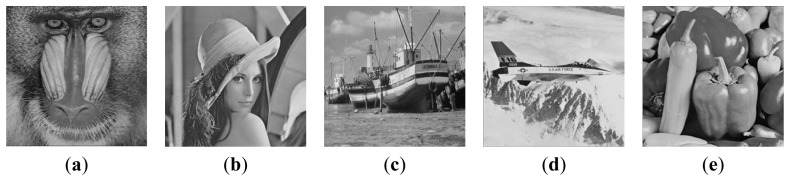
Images used for simulation experiments (**a**) Baboon. (**b**) Lena. (**c**) Boat. (**d**) Plane. (**e**) Peppers.

To evaluate the performance of the proposed method, a simulation software was developed for experimentation with real life data. In the experiments, five images each with a size of 256 × 256 pixels were used as the host images as shown in Figure 3 [42]. A passage in English was chosen as the secret text *T*. Since the recovery accuracy is defined as the ratio of the correctly recovered bytes, any text can be used as the secret text, while not influencing the experimental results significantly. The secret text *T* was transformed into a 2-dimensional array *A* by storing the bit stream of *T* in the higher bits of the elements in the transformed array *A*. Based on the number of higher bits of the elements in the transformed array *A* used for storing the bit stream of the secret text *T*, *i.e.*, the value of *m*, the hiding capacities of the secret text will be different. The transformed array *A* was encoded with the DRPE technique to hide into the expanded host images with a size of 512 × 512 pixels. For simplicity, we only performed the simulation experiments following the hiding method in reference [28].

**Figure 4.**
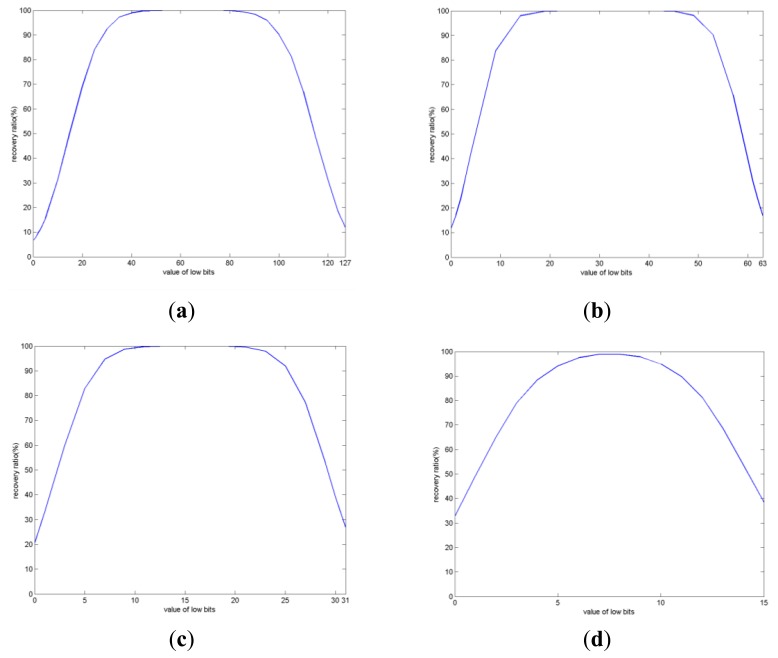
Recovery accuracies of the secret text with different methods being used to fill the lower bits of the elements in the transformed array *A*. The host image is Lena (**a**) m = 1, α = 0.02. (**b**) m = 2, α = 0.05. (**c**) m = 3, α = 0.08. (**d**) m = 4, α = 0.10.

If *m* higher bits of the elements in the transformed array *A* are used to store the bit stream of the secret text *T*, while the remaining 8 – m lower bits are filled with 0 s or 1 s, the value of the 8 – m lower bits may range from 0 to 2^8 –^ *^m^* – 1. Figure 4 shows the experimental results corresponding to the recovery accuracies of the secret text when different methods are used to fill the lower bits of the elements in the transformed array *A*. The Lena image shown in Figure 3(b) is used as the host image.

From the experimental results shown in Figure 4, it is evident that the recovery accuracy is higher when the value of the lower bits is closer to 2^7 – *m*^. Our experimentation with other host images generated similar results. The reason for such results may be explained as follows: (1) Due to computational errors existing during the procedure of encoding, embedding, extraction and decryption of DRPE based information hiding, slight difference may exist between the decrypted array *A*′ and the original transformed array *A*; (2) Since the bit stream of the secret text *T* is stored in the higher bits of the elements in the transformed array *A*, to increase the recovery accuracy, the influence of the computational errors to the higher bits of the elements in the transformed array should be decreased mostly; (3) If the value of the lower bits of the elements in the transformed array *A* is around 2^7 – *m*^, the higher bits of decrypted value will be invariant compared to those of the original value with the maximum possible, either the decrypted value is greater than or less than the original one. For example, when *m* = 2, the value of the lower bits of an element may range from 0 to 63. If we set the value of the lower bits to 32, then when the difference of the decrypted value and the original one is between −32 and 31, the 2 highest bits will be invariant. Therefore, if *m* higher bits of the elements in the transformed array *A* are used to store the bit stream of the secret text *T*. To obtain higher recovery accuracy of the secret text, it is better to set the values of the lower bits of the elements in *A* to around 2^7 – *m*^.

From Figure 4, it is evident that the number of higher bits (*m*) of the elements in the transformed array *A* being used to store the bit stream of the secret text *T* significantly influences the recovery accuracy of the secret text *T*. The less the value of *m*, the higher the recovery accuracy. The corresponding experimental results are shown in Table 2, where the values of *m* are set to 1, 2, 3, and 4, respectively. The values of the lower bits in the transformed array *A* are set to 2^7 – *m*^ to maximize the recovery accuracies of the secret text *T*. The results in Table 2 include the recovery accuracies of the secret text *T* obtained by using Lena as the host image and the average recovery accuracies obtained by using the five images shown in Figure 3 as the host images.

With greater value of *m*, the recovery accuracy of the secret text is less, especially when the values of α are identical. For a fair comparison, the values of α should be identical, since they are also related to the recovery accuracy. For example, when *m* = 1, the value of the lower bits is 64, and α = 0.02, the recovery accuracy approaches 100% by using Lena as the host image. For *m* = 2, the value of the lower bits is 32, and α = 0.02, the recovery accuracy is 91.6687% by using Lena as the host image. However, for *m* = 2, the value of the lower bits is 32, and α = 0.05, the recovery accuracy becomes 100% by using Lena as the host image. Finally, when *m* = 3, the value of the lower bits is 16, and α = 0.05, the recovery accuracy is 98.697917% by using Lena as the host image. Our experimentation with other host images yielded similar results. Therefore, when transforming the secret text *T* into the 2-dimentional array *A*, the bit stream of *T* should be stored into the higher bits of elements in *A* as much as possible.

**Figure 5.**
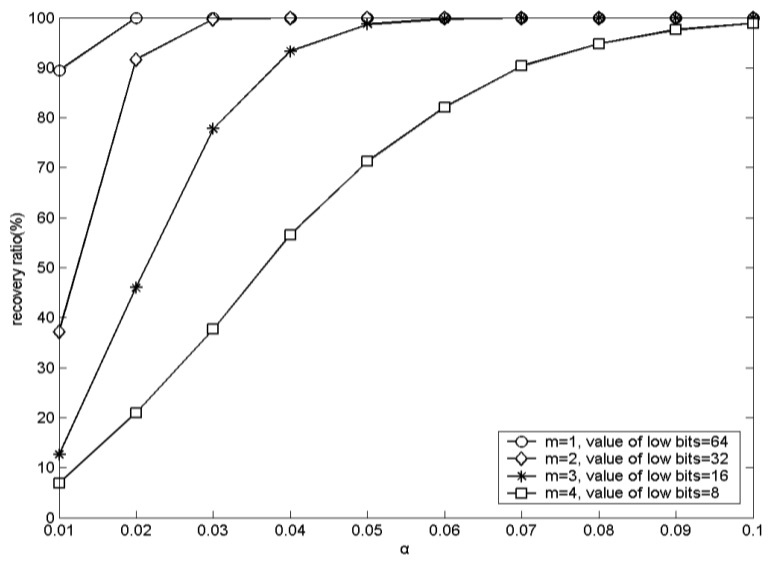
Recovery accuracies of the secret text *T* with different values of α by using Lena as the host image.

The experimental results corresponding to the recovery accuracies with different values of α by using Lena as the host image are shown in Figure 5. From Figure 5, it is evident that the greater the value of α, the higher the recovery accuracy. The experiments with other host images generated similar results. Due to the inherent characteristics of the DRPE based information hiding, with larger value of α, the decrypted array *A*′ will be closer to the original transformed array *A*. Thus, the higher bits of the elements in *A*′ will be closer to those of the elements in *A*. Therefore, to obtain higher recovery accuracy, the value of α should be as high as possible.

**Figure 6.**
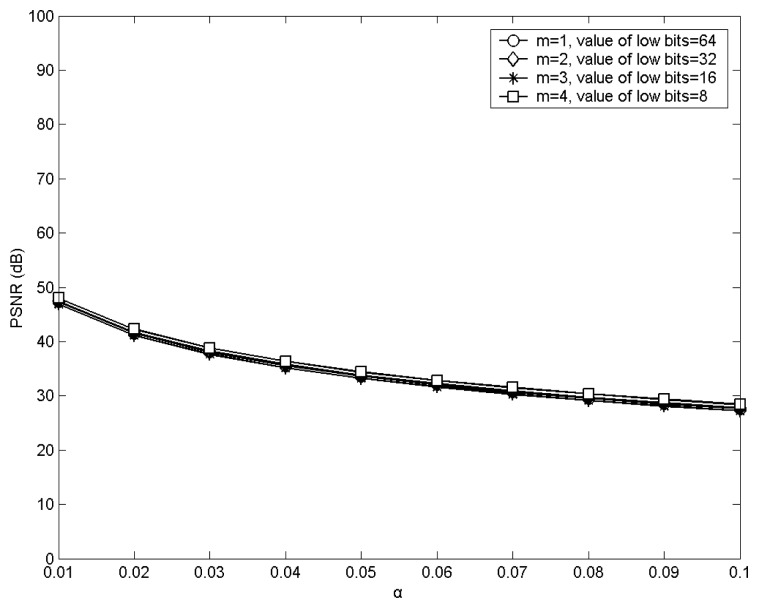
The qualities of the images embedded with hidden data with different values of α by using Lena as the host image.

The experimental results corresponding to the qualities of the images embedded with hidden data may be determined by using the peak-signal-noise-ratio (PSNR) as a performance parameter. Figure 6 depicts these results for different values of α by using Lena as the host image.

From Figure 6, it is obvious that the greater the value of α, the lower the quality of the image embedded with hidden data. Therefore, to obtain higher quality of the image embedded with hidden data, the value of α should be lower.

Based on the above mentioned results, we can infer the following:

1. To increase the hiding capacity, the value of *m* should be higher;
2. To increase the recovery accuracy, the value of *m* should be lower, while the value of α should be higher;
3. Whatever the value of *m* is, the value of the lower bits of the elements in the transformed array should be set at around 2^7 – *m*^ to obtain higher recovery accuracy;
4. To increase the quality of the image embedded with hidden data, the value of α should be lower.

Thus, to increase the hiding capacity, the value of *m* should be higher, which may decrease the recovery accuracy. To increase the recovery accuracy, the value of α should be higher, which may decrease the quality of the image embedded with hidden data. Therefore, based on the secret text to be hidden, one may adjust the values of *m* and α to tradeoff the hiding capacity, the recovery accuracy and the quality of the image embedded with hidden data. After ensuring the hiding capacity, the value of *m* may be set as low as possible to increase the recovery accuracy. For example, when the value of *m* is set to 1, if it is enough to store all of the secret text information bits, then it is not necessary to set *m* = 2 to store some secret text information bits into the second highest bits of the elements in the transformed array. After ensuring the recovery accuracy, the value of α may be set as low as possible to increase the quality of the image embedded with hidden data. For example, for *m* = 1 and α = 0.02, or *m* = 2 and α = 0.05, and the value of the lower bits is set to 2^7 – *m*^, the recovery accuracy will be 100%. Therefore, to increase the quality of the image embedded with hidden data, for *m* = 1, the value of α should not be greater than 0.02; while for *m* = 2, the value of α should not be greater than 0.05.

**Figure 7.**
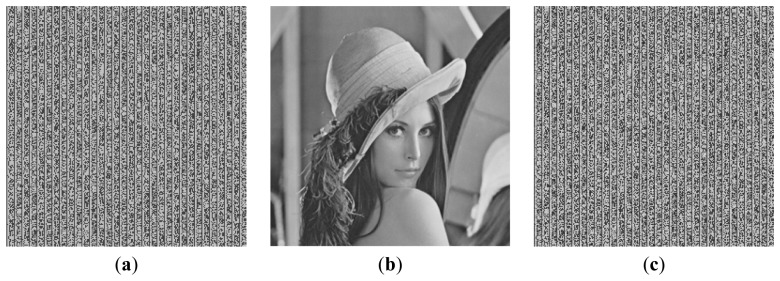
(**a**) The array transformed from the secret text with *m* = 1. (**b**) The image embedded with hidden data with α = 0.02. (**c**) The recovered transformed array. The recovery accuracy is 100%.

**Figure 10.**
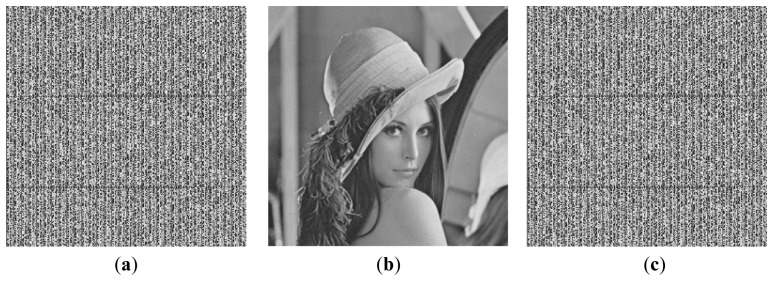
(**a**) The array transformed from the secret text with *m* = 4. (**b**) The image embedded with hidden data with α = 0.10. (**c**) The recovered transformed array. The recovery accuracy is 98.86%.

To demonstrate the above analysis and conclusions more clearly, some images obtained in the simulation experiments are shown below. Here, we show the arrays transformed from the secret text with, the images embedded with hidden data and the recovered transformed arrays. The arrays encoded with the DRPE technique are random resulting from the characteristics of the DRPE technique [2]. So, we did not show the arrays encoded with the DRPE technique. In addition, there are some parameters being used in the proposed method, such as the number (*m*) of the higher bits used to store the bit stream of the secret text, the method used to fill the lower bits in the transformed array and the value of the superimposition coefficient α. They may combine with different values, resulting in many combinations. Using image Lena as the host image, the representative results of *m* = 1, 2, 3, 4 corresponding to α = 0.02, 0.05, 0.08, 0.10, while setting the value of the lower bits of the elements in the transformed array to 2^7 – *m*^ are shown from Figure 7 to Figure 10. Since it is hard to recognize the recovery accuracy of the secret text from the image of the recovered transformed array, we also give the values of the recovery accuracies.

## 5. Conclusions

The main purpose of this paper is to apply the DRPE-based image hiding method to text encryption and hiding. In this technique, the secret text is transformed to a 2-dimensional array by storing the text bit stream in the higher bits of the transformed array. The transformed array can be viewed as an image. The DRPE-based image hiding technique is used to encode and hide the transformed array to an expanded host image.

Detailed analytical and experimental results show that: (1) the greater number (*m*) of the higher bits of the elements in the transformed array to be used for storing the bit stream of the secret text results in higher hiding capacity and lower recovery accuracy; (2) the greater value of the superimposition coefficient α results in higher recovery accuracy and lower quality of the image embedded with hidden data; (3) setting the value of the lower bits of the elements in the transformed array to approximately 2^7 – *m*^ results in the best recovery accuracy. By adjusting the values of *m* and α properly, one may achieve the optimal hiding capacity, recovery accuracy and quality of the image embedded with hidden data.

The proposed method combines the optical information processing technique by applying optical information hiding method to text encryption and hiding, which increases the security of the secret text and takes use of the advantages of optical information processing technique. In addition, it ensures acceptable hiding capacity and recovery accuracy of the secret text.

## Figures and Tables

**Figure 8.**
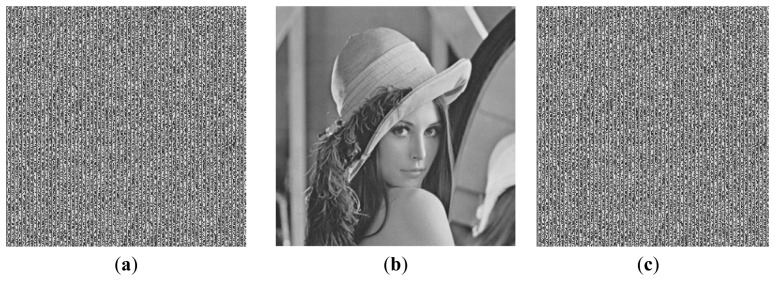
(**a**) The array transformed from the secret text with *m* = 2. (**b**) The image embedded with hidden data with α = 0.05. (**c**) The recovered transformed array. The recovery accuracy is 100%.

**Figure 9.**
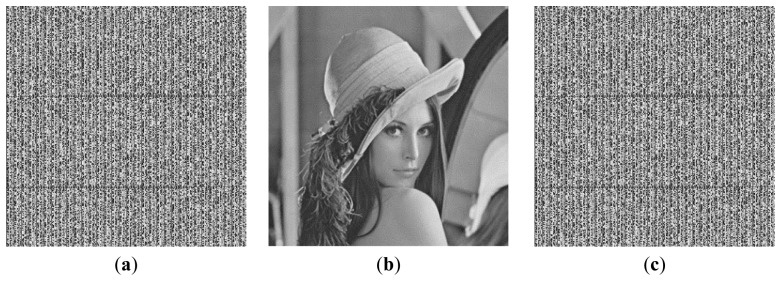
(**a**) The array transformed from the secret text with *m* = 3. (**b**) The image embedded with hidden data with α = 0.08. (**c**) The recovered transformed array. The recovery accuracy is 100%.

**Table 1.** Symbols used in the paper.

*T*	**Secret text**
*A*	2-dimentional array transformed from *T*
*k*	Secret key for DRPE encoding
*A*_1_	Array obtained by encoding *A* with the DRPE technique
*I*	Original host image
*I*_1_	Expanded host image
*I*_2_	Image embedded with hidden data by embedding *A*_1_ into *I*_1_
α	Superimposition coefficient
*A*_1_′	Array extracted from *I*_2_
*A*′	Array decrypted from *A*_1_ with the DRPE technique
*T*′	Recovered secret text

**Table 2.** Recovery accuracies of the secret text *T* with different values of *m*. The values of the lower bits are set to 2^7 – *m*^.

Value of m	m = 1			
Value of lower bits	64			
Value of α	α = 0.02		α = 0.05	
Host image	Lena	average	Lena	average
Recovery accuracy (%)	100	100	100	100
Value of m	m = 2			
Value of lower bits	32			
Value of α	α = 0.02		α = 0.05	
Host image	Lena	average	Lena	Average
Recovery accuracy (%)	91.6687	91.5759	100	100
Value of m	m = 3			
Value of lower bits	16			
Value of α	α = 0.05		α = 0.08	
Host image	Lena	average	Lena	Average
Recovery accuracy (%)	98.697917	98.2568	100	99.6997
Value of m	m = 4			
Value of lower bits	8			
Value of α	α = 0.08		α = 0.10	
Host image	Lena	average	Lena	Average
Recovery accuracy (%)	94.8029	91.7242	98.8617	94.6265
